# Engagement with health in national climate change commitments under the Paris Agreement: a global mixed-methods analysis of the nationally determined contributions

**DOI:** 10.1016/S2542-5196(20)30302-8

**Published:** 2021-02-10

**Authors:** Niheer Dasandi, Hilary Graham, Pete Lampard, Slava Jankin Mikhaylov

**Affiliations:** a School of Government, University of Birmingham, Birmingham, UK; b Department of Health Sciences, University of York, York, UK; c Data Science Lab, Hertie School, Berlin, Germany

## Abstract

**Background:**

Instituted under the Paris Agreement, nationally determined contributions (NDCs) outline countries' plans for mitigating and adapting to climate change. They are the primary policy instrument for protecting people's health in the face of rising global temperatures. However, evidence on engagement with health in the NDCs is scarce. In this study, we aimed to examine how public health is incorporated in the NDCs, and how different patterns of engagement might be related to broader inequalities and tensions in global climate politics.

**Methods:**

We analysed the NDCs in the UN Framework Convention on Climate Change registry submitted by 185 countries. Using content analysis and natural language processing (NLP) methods, we developed measures of health engagement. Multivariate regression analyses examined whether country-level factors (eg, population size, gross domestic product [GDP], and climate-related exposures) were associated with greater health engagement. Using NLP methods, we compared health engagement with other climate-related challenges (ie, economy, energy, and agriculture) and examined broader differences in the keyword terms used in countries with high and low health engagement in their NDCs.

**Findings:**

Countries that did not mention health in their NDCs were clustered in high-income countries, whereas greater health engagement was concentrated in low-income and middle-income countries. Having a low GDP per capita and being a small island developing state were associated with higher levels of health engagement. In addition, higher levels of population exposure to temperature change and ambient air pollution were associated with more health coverage included in a country's NDC. Variation in health engagement was greater than for other climate-related issues and reflected wider differences in countries' approaches to the NDCs.

**Interpretation:**

A focus on health in the NDCs follows broader patterns of global inequalities. Poorer and climate-vulnerable countries that contribute least to climate change are more likely to engage with health in their NDCs, while richer countries focus on non-health sectors in their NDCs, such as energy and the economy.

**Funding:**

This work was in part funded through an unrestricted grant from the Wellcome Trust and supported by The Economic and Social Research Council.

## Introduction

Climate change is threatening human health by irrevocably damaging the planetary systems on which life depends.^1^, ^2^, ^3^ Addressing these threats is fundamental to the United Nations Framework Convention on Climate Change (UNFCCC), the global climate governance framework designed to protect current and future generations from “dangerous anthropogenic interference with the climate system”.^4^ In line with this ambition, the UNFCCC's 2015 Paris Agreement seeks to hold the increase in global temperature to well below the critical 2°C threshold beyond which the climate system becomes increasingly inimical to public health.^2^ Achieving this ambitious goal rests on nationally determined contributions (NDCs). As of January, 2020, 185 nations had submitted their first NDCs.

NDCs are voluntary and non-binding commitments to reduce emissions made by state parties to the Paris Agreement; they are also encouraged, but not required, to include adaptation plans in their NDCs.^3^ Because the NDC regime consolidates the position of state parties as the primary agents of climate action,^4^ NDCs are the key global policy instrument for protecting the health of current and future populations. Both mitigation and adaptation are essential to protect public health.^5^, ^6^

The flexible structure of the NDCs facilitates this health focus. It enables mitigation, which is urgently required by high-emitting countries, and adaptation, which is of particular concern to low-income countries, to be aligned with public health priorities. Indeed, most NDCs do make reference to health, though with differences in the extent to which NDCs include health-related actions and plans.^2^, ^7^ Synergies between health and climate policy are especially important because health is an issue of personal and public concern;^8^ it therefore offers the potential to build public and political support for more ambitious climate change mitigation and adaptation.^9^, ^10^ Such buy-in is regarded as essential for the long-term success of the Paris Agreement. With pledges made in the 2015 set of NDCs falling well short of containing global temperature increases to below 2°C, there is an urgent need to strengthen the commitments countries make in the 2020 round of enhanced NDCs. A health framing of action on climate change could help ratchet up these ambitions.^11^

**Research in context**

**Evidence before this study**

The flexible structure of the nationally determined contributions (NDCs) enables countries to include population health in their plans for climate mitigation and adaptation. An engagement with health could therefore provide a unifying focus for countries committed to realising the ambitions of the Paris Agreement. A small number of studies have examined references to health in the NDCs and have pointed to marked national and regional variations in the prominence given to health. A larger number of social science studies have analysed the NDCs as political documents that identify the priorities of countries signed up to the Paris Agreement. However, health has not been a focus of these studies.

**Added value of this study**

Our study provides an in-depth analysis of the priority given to health in the NDCs. We then examined whether country-level factors, such as national wealth and climate-related exposures, were associated with greater health engagement in the NDCs. In addition, we compared the prominence given to health compared with other climate-related sectors (ie, the economy, energy, and agriculture) to identify further potential differences between countries. To do the analyses, we developed measures of health engagement using content analysis and natural language processing methods, which we used to identify factors associated with greater and lesser engagement with health. Overall, these findings indicate that lower-income and climate-vulnerable countries are more likely to discuss the health dimensions of climate change in their NDCs whereas higher-income countries are more likely to adopt a narrower economic perspective in their NDCs.

**Implications of all the available evidence**

Our study adds evidence for the need to prioritise health in the NDCs, which form the backbone of the Paris Agreement. In line with previous studies, we found that most countries refer to health in their NDCs. However, our study suggests that, in many NDCs, references to health are scarce and not anchored in a wider alignment between public health and action on climate change. Such evidence can help the research and policy community to appreciate that protecting and promoting people's health is not currently providing a shared and unifying global focus. Instead, engagement with health in the NDCs varies in systematic ways with wider inequalities between countries. As countries ratchet up their mitigation and adaptation ambitions, the challenge is to ensure that health does not remain the concern of climate-vulnerable regions and low-income countries but becomes a common platform for action across all countries.

The flexible structure of the NDCs also enables them to serve wider political functions. As well as being aimed at a domestic audience, they can further national interests in global negotiations (eg, by signalling countries' differential responsibility for climate change and its unequal impacts and the case for economic development and adaptation funding).^2^, ^12^ Important here is the wider structure of the UNFCCC, in which countries exert leverage through membership of negotiating groups, including the least developed countries and small island developing states (SIDS).^13^ Studies of the NDCs found that the climate mitigation and adaptation commitments of low-emitting and climate-vulnerable countries differed markedly from those of high-emitting countries.^14^, ^15^ Hence, the NDCs can be read as political texts that “reveal deeper tensions, ideas, and values about international climate policy”.^16^

Against this background, we examine how public health is incorporated in the NDCs, and how different patterns of engagement might be related to broader inequalities and tensions in global climate politics. We address this question by examining: (1) the extent and types of engagement with health, (2) the factors that are associated with different levels of health engagement and, (3) whether there are broader differences between NDCs with higher health and lower health engagement.

## Methods

### Study design

This study is based on an analysis of the NDCs submitted by 185 countries (with EU member states producing a joint NDC). We collected the NDCs from the UNFCCC registry,^17^ and examined levels of health engagement using both content analysis and natural language processing (NLP) methods. This analysis also enabled us to produce two measures of health engagement: (1) health engagement score (HES), which showed the depth of countries' engagement on the basis of the specificity and detail of health references, and (2) the health topic proportion (HTP), which measured the breadth of health engagement by measuring the weight given to a health theme in each state's NDC. We used these measures in a multivariate regression analysis to examine the country characteristics associated with different levels of health engagement. We then turned to NLP methods to analyse the differences in the content of NDCs with low and high levels of health engagement.

### Content analysis

We assessed the level of engagement with health by using content analysis of health references in the NDCs. Two coders independently identified references to health in each NDC on the basis of automated and manual searches, using search terms developed iteratively through the course of search (appendix p 1). Based on these results, each NDC was given a HES between 0 and 5, in which 0 indicated no health reference, and 5 represented the highest level of health engagement. Any mention of health allocated an entry score of 1. This score was amended on the basis of more specific or detailed references, up to a highest score of 5 (panel, table 1).

Panel **Categories of health references in the nationally determined contributions**

**Impact**

• General: included the effect of climate change on health generally or the health sector
• Specific: included the effect of climate change on specific health outcomes

**Adaptation**

• General: included the need for an adaptation plan, mentioning health, but with no further detail
• Specific: included the need for an adaptation plan, mentioning a specific health outcome, but with no further detail
• Detailed: included detailed information and plans about necessary provisions for adaptation in the area of health

**Mitigation**

• General: included a link between health and mitigation

**Co-benefits**

• General: included information about measures that would have co-benefits for health and climate change
• Specific: included co-benefit measures with information about specific health benefits

**Trade-offs**

• General: referred to mentions of financial trade-offs involved in addressing both climate change and health

**Background**

• General: referred to descriptions of a country's health profile and health challenges

**Table 1:** Types of health reference in the NDCs and their effect on initial entry HES

		**HES entry**	**Example**
Any mention		1	..
Effect			
	General	1	“Other effects [of climate change] have been observed or measured in the sectors of agriculture and human health, as well as biodiversity” (Cuba's 2016 NDC)
	Specific	3	“Examples of direct impacts include death, injury, psychological disorders, and damage to public health infrastructure” (Cambodia's 2017 NDC)
Adaptation			
	General	2	“El Salvador has resorted to sectoral adaptation strategies with an emphasis on agriculture, resources, infrastructure and health, contained in the National Strategy for Climate Change and in the Plan National Climate Change” (El Salvador's 2017 NDC)
	Detailed	3	“Minimize climate-related health risks through: strengthening integrated risk monitoring and early warning systems and response for climate sensitive diseases” (Bhutan's 2017 NDC)
Mitigation		2	“This strategy seeks to articulate and link effective energy and environmental plans by establishing goals and objectives in the area of energy, water, waste, and health for the short, medium, and long-term” (Chile's 2017 NDC)
Co-benefit			
	General	2	“Regarding mitigation… sets a clear obligation to give priority to the least costly mitigation actions, that at the same time derived in health and well-being co-benefits to the Mexican population” (Mexico's 2016 NDC)
	Specific	3	“Create a market for clean and efficient household cooking solutions in order to save lives, improve livelihoods, empower women, and protect the environment” (Lesotho's 2017 NDC)
Trade-off		2	“As a developing country, the lack of fiscal space to finance priority issues including poverty reduction policies including investments in education, health, and basic infrastructure constrains the country's effort to finance and implement climate mitigation and adaptation policies” (Ghana's 2016 NDC)
Background		1	“Equatorial Guinea is a developing country, whose economy depends exclusively on extractive industries and has to face a series of development challenges, such as: poverty, education, health, road infrastructure, etc” (Equatorial Guinea's 2018 NDC)

HES=health engagement score. NDC=nationally determined contribution.

This HES measure showed the depth of countries' engagement with health (table 1). It is worth noting that although the framework distinguished between mitigation and co-benefits, they were closely connected in that co-benefit referred to mentions of mitigation measures that had a health co-benefit. We provide a more detailed discussion of the scoring framework with examples of different types of health mentions in the appendix (pp 1–3).

### NLP

We also used NLP methods, which have been applied more widely to political texts to examine the level of government engagement and position on various issues in world politics.^18^ We used two different NLP methods. The first was an application of a version of the frequently used Latent Dirichlet Allocation probabilistic topic model to the NDC corpus.^19^ Probabilistic topic models are algorithms for discovering the main themes in large, unstructured collections of textual data and have been widely applied to genetic and medical data, images, and social networks. Here, we used the keyword assisted topic model (keyATM), which allowed us to provide the topic model with a small number of keywords to improve topic identification and labelling, as well as to enhance the stability and reproducibility of the model (appendix p 5).^20^ We identified keywords in the corpus of NDC documents associated with four themes (ie, health, economics, energy, and agriculture), which were used in the keyATM to produce six topics (consisting of four topics that were based on specified themes and two residual topics that captured any remaining semantic content). We used the proportion of the health topic in each NDC document as an alternative measure of health engagement (ie, the HTP). We provide a more detailed discussion of the keyATM topic model analysis and the HTP measure in the appendix (pp 5–10).

The second NLP method was based on the concept of keyness, a concept used in text analysis to indicate that particular words and phrases reflect important themes within a document, and can be used to uncover the principal differences between groups of documents.^21^ Specifically, we identified the terms that were statistically most distinct in NDCs with higher health engagement than NDCs with lower health engagement. Hence, the keyness analysis showed broader differences in the content of NDCs with a higher HES than those with a lower HES.

### Statistical analysis

We examined the factors associated with higher levels of health engagement in the NDCs using a multivariate regression analysis. We used three different outcome variables to measure health engagement: (1) any reference to health, (2) HES, and (3) the HTP. We used a logistic regression for the first, and ordinary least squares regressions for the latter two. We provide results from additional analyses using alternative outcome variables (eg, the total count of health terms in the NDCs) to do sensitivity analyses of our findings in the appendix (pp 12–14).

We included several country co-variates in our regression models linked to political and economic factors, as well as variables associated with climate change and health, which could influence health engagement. The selection of variables is discussed further in the appendix (pp 11–12). These variables included the size of a country's population, gross domestic product (GDP) per capita, and level of democracy. Previous studies found that SIDS lead engagement on climate change and health in multilateral fora,^22^ so we included a dummy variable for whether a country belonged to a SIDS. Our climate change and health variables included health spending (measured as % of GDP), coal rents (measured as % of GDP) as a measure of the importance of fossil fuel revenues for the national economy, population-weighted change in a country's temperature, and exposure to air pollution. The last two variables relate directly to the connection between health and climate change.

The data for most of these variables came from the World Bank's world development indicators.^23^ The democracy measure combined freedom house and polity scores,^16^ the air pollution measure was from WHO's data for average country exposure to ambient air pollution based on concentrations of fine particle matter (PM_2·5_),^4^ and the population-weighted temperature change variable, which measured average temperature change experienced by human populations, was from the report of the *Lancet* Countdown on Health and Climate Change.^24^ We used data from 2016, as most NDCs were published in that year, and fill any missing observations with data from the years 2014 or 2015. As data were unavailable for some countries, the sample size in the regression analysis was 175. Summary statistics are provided in the appendix (p 14).

### Role of the funding source

The funder of the study had no role in study design, data collection, data analysis, data interpretation, or writing of the report. All authors had full access to all the data in the study and had final responsibility for the decision to submit for publication.

## Results

**Figure 1:**
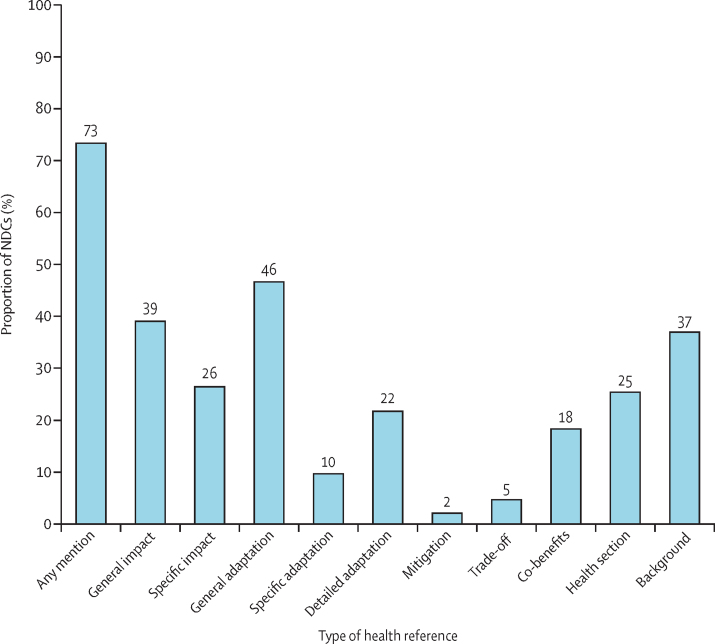
Proportion of NDCs using different types of health reference, based on content analyses of nationally determined contributions NDC=nationally determined contribution.

Most NDCs (135 [73%] of 185 countries) include a reference to health. However, there were significant differences in the extent of health engagement (figure 1). For example, 94 (51%) countries made only a brief general reference to health or did not mention health at all. Examples of limited engagement can be seen in the NDCs of countries such as Saudi Arabia, South Korea, and Switzerland. South Korea, for instance, made only a brief single reference to strengthening adaptation “for the management of the negative impacts of climate change on health”.^25^ NDCs from 60 (32%) countries specifically mentioned health and noted specific climate-related health outcomes or adaptation measures (eg, Argentina, Bangladesh, and Canada), 40 (22%) countries' NDCs had detailed health adaptation plans (eg, Burkina Faso, India, and Vanuatu). Therefore, the NDCs also varied in terms of the types of health references included (figure 1). The most frequent type of health reference was to the general adaptation of climate change on health, which was included in NDCs from 86 (46%) countries. Little over half of NDCs (51%; 95 countries) referred to health adaptation (general or specific), and NDCs from 34 (18%) countries referred to the health co-benefits of climate action, including Antigua, Cameroon, and Mexico.

**Figure 2:**
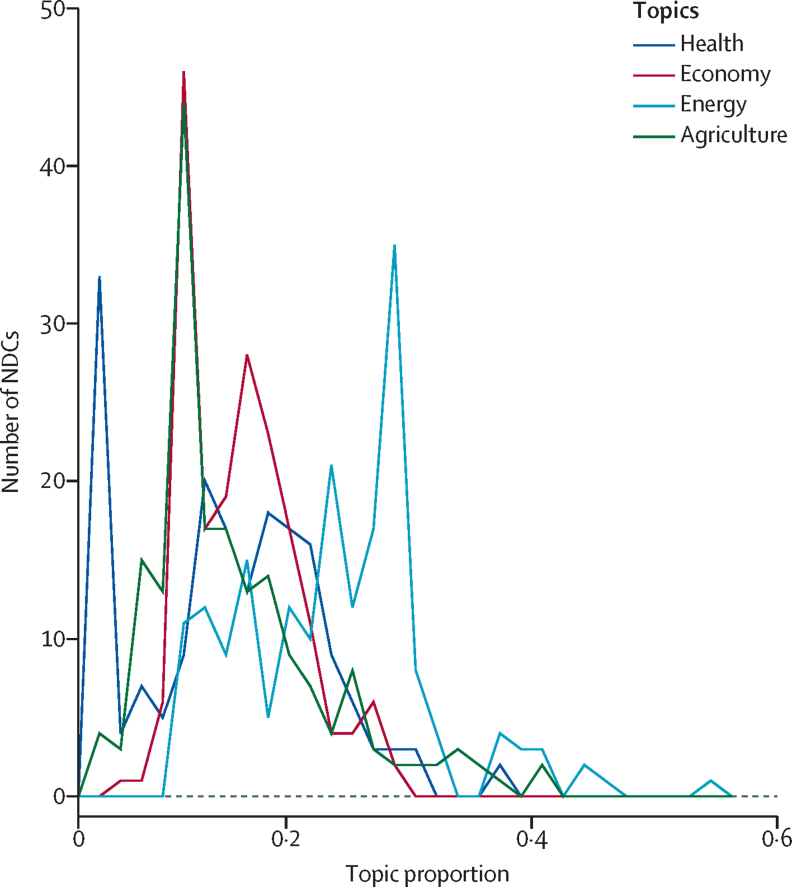
Frequency polygon of topics in NDCs, based on topic model analysis NDC=nationally determined contribution.

To examine how engagement with health in the NDCs compared to other topics, we used topic model analyses. Figure 2 shows the total number of NDCs with different topic proportions for each of the four topics (ie, health, economy, energy, and agriculture). For health, we found that the highest number of NDCs had a very low topic proportion, further showing that most NDCs make only a brief reference to health. By contrast, we found that many NDCs had high topic proportions for agriculture and economy, and energy was discussed most in the NDCs. Figure 2 also indicates that there is much higher variation in the topic proportion for health than the other three topics. This variation in health engagement can also be seen in a density plot of the four topics, which is presented in the appendix (p 7), in which the area under the curve is normalised for each topic, enabling more direct comparisons. The density plot further shows the higher variation in how much countries discuss health than with other topics. The density plot also shows that fewer countries discuss health in the same level of detail as other topics, and that countries devote a higher proportion of their NDCs to discussing energy than for other topics.

**Figure 3:**
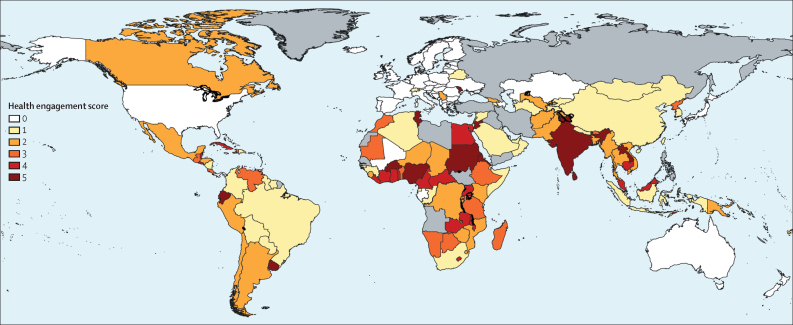
Health engagement scores by country Grey indicates countries that are either not parties to the Paris Agreement or have not submitted an NDC as of January, 2020. NDC=nationally determined contribution.

The marked variation in health engagement across NDCs can be seen when differences in countries' HES were considered (figure 3). Figure 3 shows a divide between high-income countries that had no engagement or lower levels of engagement with health in their NDCs and low-income and middle-income countries that had higher levels of health engagement in their NDCs. The countries that made no mention of health were predominantly high-income countries, including Australia, the EU member states, Japan, New Zealand, and the USA. By contrast, most countries in Africa, Asia, and Latin America made some reference to health in their NDCs. Figure 3 also shows the degree of health engagement based on the HES, showing that countries in south Asia, sub-Saharan Africa, and parts of southeast Asia have higher levels of health engagement than other countries analysed. SIDS also had higher HES compared with other countries; however, most are not visible on the map because of their small size. We provide additional analysis of the distribution of HES across the NDCs in the appendix (pp 3–4).

To further investigate the patterning of HES and topic proportion, we considered the factors associated with health engagement in the NDCs using a multivariate regression analysis. Results of the regression analysis looking at country characteristics associated with health engagement in the NDCs are shown in table 2. The first model presents the results of a logistic regression that examined the factors that were associated with any mention of health in the NDCs. The second and third models used an ordinary least squares regression analysis to examine factors linked to high levels of engagement with health, which were based on two alternative outcome variables, the HES and the HTP.

**Table 2:** Association between country characteristics and health engagement in NDCs using logistic regression and OLS regression with heteroscedasticity-robust standard errors

	**Any mention of health (logistic regression)**		**Health engagement score (OLS regression)**		**Health topic proportion (OLS regression)**	
	Coefficient (95% CI)	p value	Coefficient (95% CI)	p value	Coefficient (95% CI)	p value
Population (logged)	0·2 (−0·1 to 0·5)	0·19	0·05 (−0·1 to 0·2)	0·43	−0·002 (−0·01 to 0·003)	0·43
SIDS	3·6 (1·5 to 5·7)	0·001	0·9 (0·2 to 1·5)	0·010	0·1 (0·1 to 0·1)	<0·0001
GDP per capita (logged)	−0·5 (−1·0 to −0·1)	0·018	−0·5 (−0·6 to −0·3)	<0·0001	−0·02 (−0·02 to −0·01)	<0·0001
Democracy	−0·1 (−0·4 to 0·1)	0·33	0·01 (−0·09 to 0·1)	0·81	−0·002 (−0·01 to 0·001)	0·22
Health expenditure (% of GDP)	−0·1 (−0·3 to 0·1)	0·32	−0·02 (−0·1 to 0·1)	0·70	−0·003 (−0·006 to 0·000)	0·052
Coal rents (% of GDP)	−0·1 (−1·3 to 1·1)	0·86	−0·5 (−0·8 to −0·2)	0·0041	0·01 (−0·01 to 0·03)	0·27
Temperature change	0·7 (−0·5 to 1·9)	0·26	0·6 (−0·04 to 1·1)	0·066	0·03 (0·01 to 0·05)	0·016
Air pollution exposure	0·1 (−0·002 to 0·1)	0·059	0·01 (−0·01 to 0·03)	0·23	0·001 (0 to 0·001)	0·033
Pseudo-R^2^	0·6	..	..	..	..	..
R^2^	..	..	0·3	..	0·5	..
N	175	..	175	..	175	..

GDP=gross domestic product. NDC=nationally determined contribution. OLS=ordinary least squares. SIDS=small island developing states.

In the first model in table 2, there were two factors that had statistically significant relationships to whether a country mentioned health in its NDC. These were whether the country is a SIDS (3·6, 95% CI 1·5 to 5·7), and a country's level of GDP per capita (–0·5, −1·0 to −0·01), showing that SIDS have a higher likelihood of having NDCs that refer to health compared with countries that are not SIDS, and that richer countries are less likely to mention health in their NDCs than poorer countries. These results indicate that exposure to ambient air pollution has a positive relationship with mentioning health in NDCs; however, this factor falls outside the 95% CI (p=0·062). Population size, level of democracy, health expenditure, coal rents, and population-weighted temperature change were not associated with whether a country mentioned health in its NDC.

Table 2 also presents the results of the ordinary least squares regression analysis, which examined—using the HES and HTP measures—the factors that were associated with health engagement. Our results also indicated that SIDS and GDP per capita have statistically significant associations with both measures of health engagement (ie, HES and HTP). The higher a country's GDP per capita, the less it engages with health, indicating that income is closely related to engagement with health in NDCs.

Our results suggest that additional factors might be related to health engagement, although these are statistically significant for either HES or HTP, rather than for both. Coal rents (measured as % of GDP) had a negative effect of −0·5 on the HES (95% CI −0·8 to −0·2), suggesting that countries with higher coal revenues were less engaged with health in the NDCs than countries with low coal revenues. Greater temperature change and exposure to air pollution were associated with more discussion of health in countries' NDCs. The population-weighted temperature change had a statistically significant positive effect of 0·02 on the HTP (p=0·016, p=0·066 for HES). When we used the total count of health terms as an outcome variable (appendix p 13), we also found that temperature change had a significant effect. Our results showed that high exposure to ambient air pollution was positively associated with HTP, though not with HES. Population size, level of democracy, and health expenditure were not related to health engagement with either measure. The results of the sensitivity analysis broadly supported the findings here; in particular providing further evidence that GDP per capita is negatively correlated with health engagement.

**Figure 4:**
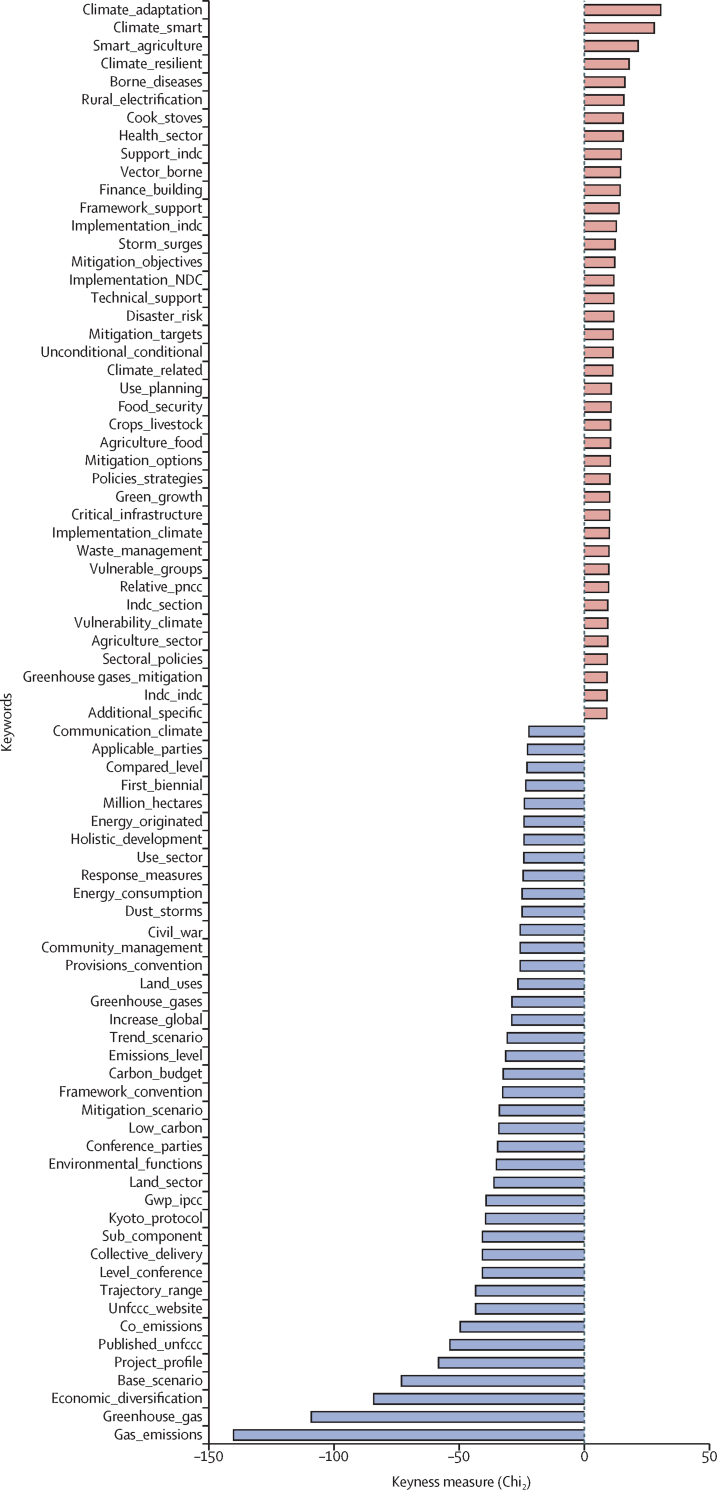
Keywords distinguishing countries with low health engagement and those with higher health engagement Bigram of statistically distinct words in NDCs for health engagement scores ranging from 0 to 1 (shown in blue) versus scores ranging from 2 to 5 (shown in red). GWP=global warming potential. indc=intended nationally determined contribution. NDC=nationally determined contribution. PNCC=national policy on climate change. UNFCCC=UN Framework Convention on Climate Change.

We then considered whether differences in levels of health engagement reflected wider dissimilarities in the content of countries' NDCs. In other words, we examined the more general differences in the approach taken in NDCs that had high levels of health engagement than those with low health engagement. We did this through the NLP keyness analysis. The terms in the NDCs that were statistically most likely to appear in the NDCs with low levels of health engagement compared with terms most likely to appear in NDCs with high levels of engagements are shown in figure 4. The most statistically distinct terms appeared in NDCs with low levels of engagement with health, with a HES between 0 and 1, compared with the most statistically distinct terms that appeared in the NDCs with a HES between 2 and 5. We did an additional keyness analysis that compared the differences in the content of NDCs with high and low engagement with health in the appendix (pp 14–17).

The keywords identified in the keyness analysis were closely related to issues included in the Paris Agreement.^26^ However, there were significant differences in the aspects of the Paris Agreement that countries with low health engagement emphasised in their NDCs compared with countries with high engagement. Our results indicated that NDCs with no or low levels of health engagement had a narrower focus on emissions, energy use, and the UNFCCC framework than countries with higher levels of health engagement mentioned in their NDCs with terms such as greenhouse_gas, emissions_level, base_scenario, and energy_consumption (figure 4; appendix pp 15–16). The emphasis on the Paris Agreement was shown by terms such as level_conference and kyoto_protocol. These NDCs also made greater reference to economic factors (eg, economic_diversification and gdp_growth) than NDCs that did not emphasise health.

By contrast, NDCs with higher levels of health engagement tended to have a much broader focus on issues related to climate change mitigation and adaptation included in the Paris Agreement (eg, climate_adaptation, climate_resilient, mitigation_adaptation), as well as greater reference to health-related domains affected by climate change. Thus, in addition to health, we found keywords that were linked to agriculture and food (eg, smart_agriculture and food_security), water and waste, and references to rural_electrification, disaster_risk, and urban_development were associated with NDCs that had high levels of health engagement. Another important feature of NDCs with higher health engagement was their emphasis on international finance and technology transfer, included in articles 9–11 of the Paris Agreement, which were indicated by terms such as finance_building, technology_building, and conditional_unconditional (which referred to plans that were conditional on external funding).

## Discussion

Our study showed that, although most NDCs mention health, there is considerable variation in the types and depth of this engagement. We also found that this variation in countries' engagement with health is much greater than for other topics, such as the economy or energy. This variation is largely explained by differences in countries' income levels, whereby the NDCs of poor, low-emitting countries (particularly SIDS) engage much more with health in their NDCs compared with richer, high-emitting countries, which make little or no mention of health in their NDCs. Furthermore, we found some evidence that countries that are more exposed to the effects of climate change (eg, countries that have populations that are experiencing greater temperature change) engage more with health, while countries that receive higher coal rents engage less with health in their NDCs.

We also found that these differences in health engagement are grounded more broadly in different approaches to the NDCs. Countries that engage less with health in their NDCs tend to have a narrower focus on the objectives set out in the Paris Agreement, and a more economic-centred approach. By contrast, countries that engage more with health in their NDCs embrace the Paris Agreement's broader societal perspective on climate effects, mitigation, and adaptation. These countries also emphasise the need for international funding and technology transfers to meet the goals of the Paris Agreement. These differences are consistent with differences noted elsewhere in the weight that higher-income and lower-income countries give to adaptation, sustainable development, and climate finance in their NDCs.^27^, ^28^

Our study adds a health perspective to these earlier studies. As an issue that resonates strongly with the public, references to health in the NDCs could strengthen commitment to climate action at a national and global level. However, our study suggests that health is being mobilised in ways that reflect existing divides in global climate politics. It is the poorest and most climate-vulnerable countries that emphasise the health dimensions of climate change in their NDCs, and do so in part to remind wealthy nations of their commitments in the Paris Agreement, particularly providing financial and technological resources to low-income countries. Thus, low-income countries, such as Comoros and Ethiopia, explicitly connect the health effects and adaptation plans of climate change in their NDCs to call for wealthier nations to provide financial and technological assistance. By contrast, there was little engagement with health in the NDCs of richer, high-emitting states, with countries such as Australia, EU member countries, and the USA making no reference at all to health in their NDCs. Very few governments of high-income countries discuss health adaptations plans in their NDCs, although they published detailed national health adaptation plans elsewhere.^29^

Our study has several limitations. First, as we have noted in the methods section, the EU member states submitted a single joint NDC. In our analysis, we assess the individual EU member states separately; however, this approach assumes that all 28 countries (the UK was included in the EU's first NDC) have an identical position on approaches to climate change adaptation and mitigation, which is unlikely to be the case. Hence, because of the submission of a single NDC for the EU, we were unable to capture potential variations in health engagement across the member states. Second, for several variables included in our regression models, there were missing data for some countries, which meant that these countries were excluded from the regression analysis, reducing our sample size to 175. These countries were either low-income countries (eg, Eritrea and Somalia) or small states (eg, Grenada), which introduced some potential bias into our analysis. Third, as we have discussed, the HESs developed in our analysis were based on the manual content analysis of health references in the NDCs, which were scored according to the framework we developed (appendix pp 1–3). This approach introduced a degree of subjectivity in interpreting and scoring each health reference, which we have sought to minimise by using a detailed scoring framework and ensuring that two coders independently scored each NDC. Lastly, our analysis included the application of probabilistic topic models to the NDC corpus. As we have discussed, we have sought to address the potential unreliability of this approach through the use of a keyATM, which has been shown to yield more stable and reproducible results. However, some degree of variability in the results remains. Despite these limitations, the results of our analysis produce strong evidence that poorer and more climate-vulnerable countries emphasise the health effects of climate change in their NDCs, while wealthy, high-emitting countries make little or no reference to health.

These findings show that engagement in the health dimensions of climate change in the first round of NDCs is an indication of a country's broader response to climate change, and reflects deeper inequalities across the world. From this perspective, the lack of engagement with health by wealthy, high-emitting nations is part of a more general effort to frame climate change as an issue that can be addressed without far-reaching political action and resource transfers from richer to poorer nations.^30^ This global disparity of commitment to health in the NDCs also means that, as countries bolster their NDCs, there is a risk that health is again side-lined by higher-income countries, with lower-income and climate-vulnerable countries left to draw attention to the large health effects of climate change that many countries experience. Health, an issue of concern for people across the world, is not currently providing a shared and unifying focus in the major instrument of global climate action. In this regard, the COVID-19 pandemic has provided a stark reminder that global health challenges do not respect national boundaries. As such, the experience of the COVID-19 pandemic could offer an important opportunity to reintegrate health into the politics of climate change in ways that can motivate global cooperation and ratchet up countries' climate change ambitions.

## Data sharing

All data and replications materials for this study are made publicly available via the Harvard Dataverse. The data and replication material are available at https://doi.org/10.7910/DVN/SSZB5I.
