# Supplementary material for: Engagement with health in national climate change commitments under the Paris Agreement: a global mixed-methods analysis of the nationally determined contributions
Source: Lancet Planet Health. 2021 Feb 10;5(2):e93–e101. doi: 10.1016/S2542-5196(20)30302-8 (PMC7887662; doi:10.1016/S2542-5196(20)30302-8)
Supplement: Supplementary appendix [file mmc1.pdf]

THE LANCET
Planetary Health

Supplementary appendix

This appendix formed part of the original submission and has been peer reviewed. We post it as supplied by the authors.

Supplement to: Dasandi N, Graham H, Lampard P, Jankin Mikhaylov S. Engagement with health in national climate change commitments under the Paris Agreement: a global mixed-methods analysis of the nationally determined contributions. *Lancet Planet Health* 2021; **5:** e93–101.

Engagement with health in national climate change commitments under the Paris Agreement

Supplementary Appendix

Content Analysis and HES

The content analysis of health engagement in the NDCs was conducted in several stages. First, we collected NDCs from the UNFCCC registry.[i] Second, two independent coders identified each reference to health. To do this, we used an initial list of health search terms taken from *the Lancet Countdown on Health and Climate Change* analysis of political engagement in the UN General Assembly[ii], and incorporated additional health terms based on our reading of the NDCs. Therefore, the list of search terms was developed iteratively using the initial list of search terms and then including any additional health terms that we identified by manually going through all of the NDCs. We provide the full list of search terms in Table S1. This list of key health search terms was also used to conduct an automated search of health terms to ensure we had captured all references in the NDCs.

Table S1. List of health search terms.

**Health Search Terms**	
health	mortality
healthcare	mortalities
diarrhoea	morbidity
diarrhea	nutrition
diarrhoeal	illness
diarrheal	illnesses
infection	ncd
disease	ncds
diseases	nutrition
virus	malnutrition
sars	malnourishment
measles	mental disorder
pneumonia	mental disorders
epidemic	stunting
epidemics	medical
pandemic	loss of life
pandemics	loss of lives
epidemiology	death
Dengue	deaths
Malaria	killed
wellbeing	HIV
well-being	AIDS
waterborne	STI
vectorborne	immunization

Third, each reference was coded independently by two coders according to the framework, which was developed iteratively and through dialogue between the two coders, each of whom read through all NDCs. If disagreement over a final HES occurred between the coders, the proportion of words given to health in the particular NDC could be used (calculated based on words within sentences co-occurring with a health term). The framework distinguishes between *general* and *specific* references, as well whether *detailed* adaptation plans are included. *General* references were those that pointed to climate change having an impact on the health sector or mentioned the need for health adaptation with no further information provided. *Specific* references include those that link climate change to more precise health outcomes relevant to the particular country or identify health adaptation measures. *Detailed* adaptation refers to a more comprehensive discussion of the actions the government will take to address the health impacts of climate change in the country. We provide examples of general, specific, and detailed references below:

El Salvador (general): *As previously noted, water resources, agriculture, road infrastructure, health and tourism development are being increasingly affected by climate change, so It is essential to define a strategic framework for action that allows identifying and putting Priority actions for adaptation to climate change are underway in these sectors.*

El Salvador (specific): *It also severely impacts and in various ways to the health sector, relating to different types of diseases, including morbidity heat stress mortality, acute diarrheal diseases (EDAs) from aggravated water pollution due to flooding, acute respiratory infections (ARIs) and vector diseases, aggravated also due to high temperatures.*

Sri Lanka (general): *In addition, impacts of climate change appear to have significant repercussions on health of the citizens and human settlements of the country*

Sri Lanka (specific): *Spread of vector borne diseases into new areas with changing patterns of local climate is a potential health hazard that needs to be allocated close attention.*

Barbados (general): *The sectors identified as most vulnerable to climate change are agriculture, fisheries, tourism, water, human health, coastal resources and human settlements.*

Barbados (specific): *Barbados will face indirect climate-related impacts including drought, flooding, and storms (physical damage), increased pest outbreaks, the spread of invasive species, the increased probability for the occurrence of vector borne and heat related illnesses and the destruction of key ecosystems which all threaten national productivity and may undermine the potential for real growth.*

Malaysia (detailed): [In separate health adaptation section] *During the Tenth Malaysia Plan, Malaysia spent over RM 9 billion on the health sector, with over RM 1.5 billion being for adaptation of the health sector to climate change. In particular, vector-borne diseases such as dengue and malaria are expected to be further exacerbated by rising temperatures and high rainfall… The control and prevention of dengue transmission using early test kits and community behavioural intervention as well as exploratory research on alternative medicines are among the efforts being undertaken… In addition, climate-related disasters are already posing huge challenges to the public disaster management systems. Ensuring clean water supply and optimal sewerage services are particularly difficult during disasters such as flooding, as experienced during the recent year-end massive flooding that affected several states, giving rise to food and water-borne diseases.*

The framework includes six types of health reference: *impact*, *adaptation*, *mitigation*, *co-benefit, trade-off*, and *background*. These refer to mentions of the impact of climate change on health, health being linked to the need to mitigate, or measures that would have co-benefits for health and climate change. The trade-off category refers to mentions of financial trade-offs involved in addressing both climate change and health. Some NDCs also included *background* health information to provide context, rather than in the discussion of their contribution itself. We assessed whether the NDC included a separate section or subsection on health. We provide examples of these different types of references below (excluding section/sub-section).

Barbados (impact): *Barbados will face indirect climate-related impacts including drought, flooding, and storms (physical damage), increased pest outbreaks, the spread of invasive species, the increased probability for the occurrence of vector borne and heat related illnesses and the destruction of key ecosystems which all threaten national productivity and may undermine the potential for real growth.*

Ghana (adaptation): *Manage climate-induced health risks; strengthen climate related disease surveillance in vulnerable communities in 3 Districts. Adopt climate change informed health information systems including traditional knowledge on health risk management.*

Gambia (mitigation): *The Republic of The Gambia includes two unconditional mitigation options in its INDC: Firstly, the use of renewable energy sources in lighting, communication and health facilities…*

Mexico (co-benefits): *Actions to abate SLCPs simultaneously contribute to climate change mitigation in the near term and to the immediate improvement of air quality, as well as to generate positive impacts on human health and ecosystems conservation.*

Dominican Republic (trade-off): *The Dominican Republic is a middle-income country, however, it faces a number of development challenges, such as: poverty, education, health, security, etc., that exacerbate the challenge of adaptation and decoupling emissions from the economy.*

Afghanistan (background): *Over the past 13 years notable achievements have been made in the areas of environment, agriculture, health, education, infrastructure, the economy, and the provision of other important basic services.*

Based on each of these individual references, each NDC was scored independently by two coders. Within this framework, no mention of a health-related term returned '0'. *General* mentions of health, the need for adaptation in the health sector, or co-benefits entered as a '1' on the scorecard. *Specific* health impacts, adaptation needs or co-benefits, such as with regard to diseases or heat-related illness, scored a '2'. Two or more specific mentions

received a ‘3’. *Detailed adaptation* plans entered at ‘3’ on the scorecard. Detailed adaptation plans, combined with specificity elsewhere received ‘4’. NDCs containing a dedicated health section or subsection were increased by ‘1’ on the scorecard. Background information regarding health (e.g. recent progress in health sector) was scored in accordance with its generality or specificity, as outlined above. On a couple of occasions, health in relation to mitigation and enforced financial trade-offs between health and other sectors were described; these were dealt with on a case-by-case basis. On the basis of this scoring framework, each NDC was independently scored by each of the coders and given a 0-5 HES. Inter-coder agreement for overall scores exceeded 90%, and the coders discussed each NDCs were there were different scores given to come to an agreement on the final HES. Figure S1 presents the total count of NDCs by the HES, showing the variation in health engagement across the NDCs.

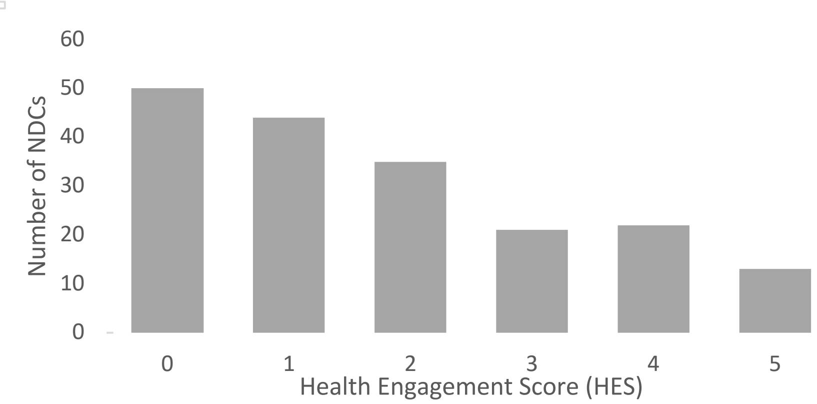

Figure S1. Count of NDCs by Health Engagement Score (HES).

Figures S2-S5 provide additional breakdowns of the NDCs according to the content analysis and the HES. The figures further demonstrate that lower income countries engaged more with health overall, and in more depth, than richer countries. Furthermore, they show that countries in African and SE Asian regions had highest engagement with health, while Europe and the Eastern Mediterranean region had lowest engagement.

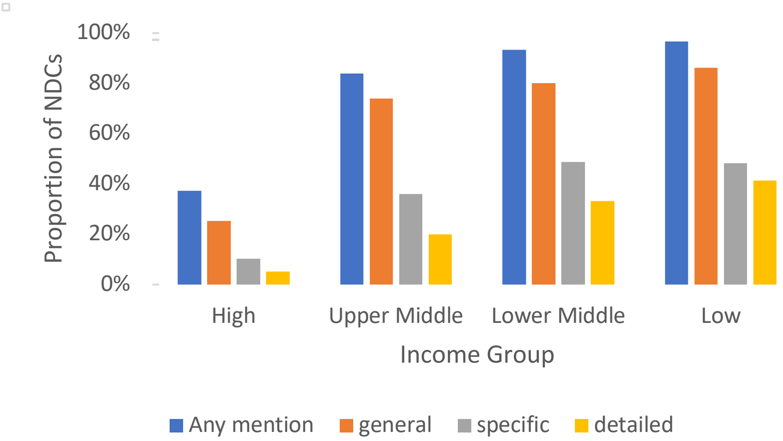

Figure S2. Proportion of NDCs by WB Income Group using different types of health reference (any mention, general, specific, and detailed) (general include general mention and general adaptation; specific includes specific mention and specific adaptation).

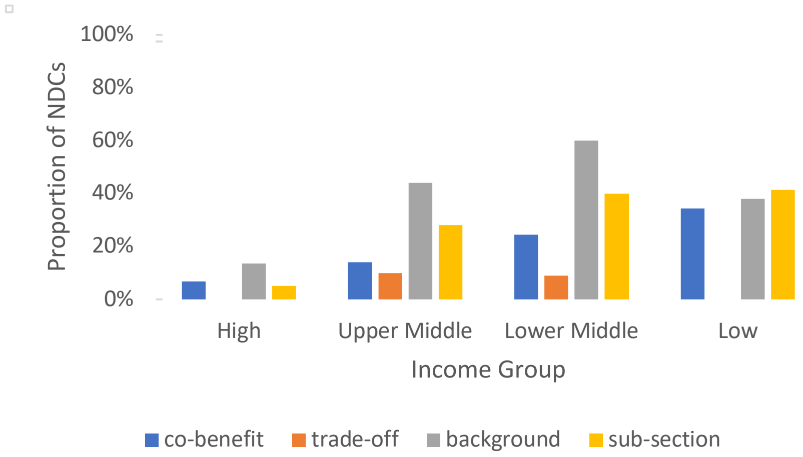

Figure S3. Proportion of NDCs by WB Income Group using different types of health reference (co-benefits, trade-offs, background information, and a health subsection)

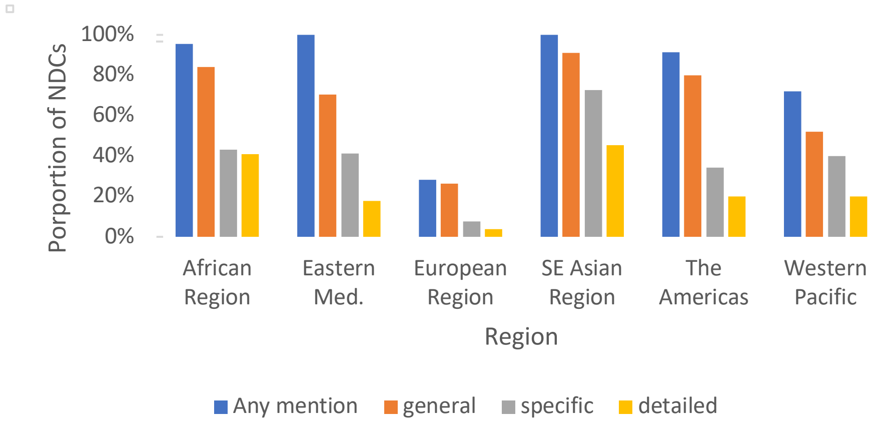

Figure S4. Proportion of NDCs by WHO Region using different types of health reference (any mention, general, specific, and detailed) (general include general mention and general adaptation; specific includes specific mention and specific adaptation).

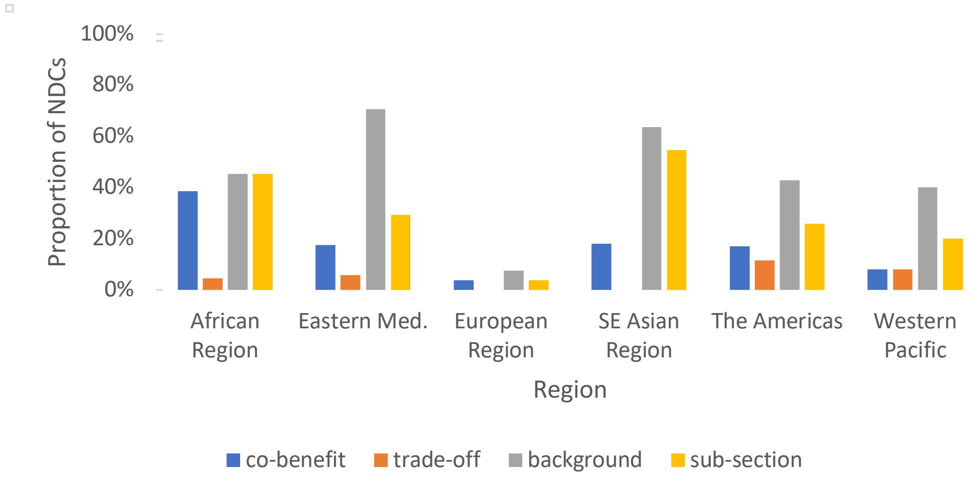

Figure S5. Proportion of NDCs by WHO Region using different types of health reference (co-benefits, trade-offs, background information, and a health subsection).

Topic Model Analysis

We employed two different NLP methods. The first was an application of a version of the popular Latent Dirichlet Allocation probabilistic topic model to the NDC corpus.[iii] Probabilistic topic models are algorithms for discovering the main themes in large unstructured collections of textual data. Here, we use a version of probabilistic topic models - the keyword assisted topic model (keyATM) [iv,v], which allows us to seed the topics with a small number of keywords to improve topic identification and labelling. Applications of the keyATM have demonstrated results superior to the standard LDA approach in terms of topic identification and reproducibility.[vi] Based on our initial reading of the NDCs, we selected four themes: health, economy, energy, and agriculture. For each topic, we produced a list of associated search terms. For the health topic, this was a truncated version of the health terms provided in Table S1. For the other three topics, we produced a list of keywords. This was based on words commonly associated with each topic, as well as words identified in the NDCs. The list of search terms for each topic can be seen in Table S2 – they are the words with the ticks next to them in each topic.

Table S2. Key words for all topics in keyATM analysis. Ticks indicate search terms used for each topic.

1	1_health	2_economy	3_energy	4_landagriculture	Other_1	Other_2
2	**illness [✓]**	economists [✓]	got [✓]	nadf [✓]	summary_energy	cordex
3	**sti [✓]**	fdi [✓]	green_energy [✓]	ecologists [✓]	emissions_showing	wintertime
4	**mortalities [✓]**	growth_model [✓]	coal_based [✓]	genetics [✓]	showing_relative	introduction_extensive
5	**ncd [✓]**	economic_damage [✓]	energy_industries [✓]	genotypes [✓]	gdp_anticipated	extensive_development [2]
6	**infection [✓]**	unemployed [✓]	energy_saving [✓]	grains [✓]	lake_majority	linked_disasters
7	**diarrhea [✓]**	aids [1]	thermal [✓]	food_supplies [✓]	population_relies	stability_food
8	**killed [✓]**	imf [✓]	energy_efficient [✓]	soil_testing [✓]	indirectly_available	threat_despite
9	**immunization [✓]**	morbidity [1]	electrification [✓]	diarrhoea [1]	developing_rapidly	despite_remain
10	**diarrhoeal [✓]**	unemployment [✓]	power_plants [✓]	waterborne [1]	strategies_leds	allow_develop
11	**diarrheal [✓]**	economies [✓]	electricity_generation [✓]	cropping [✓]	make_rapid	legal_assistance
12	**epidemic [✓]**	invest [✓]	grid [✓]	sustainable_agriculture [✓]	warming_therefore	recognizes_countries
13	**healthcare [✓]**	wealth [✓]	demand [✓]	natural_resource [✓]	heads_allow	lowering_maintaining
14	**hiv [✓]**	economically [✓]	fuel [✓]	resistant [✓]	result_ghg	maintaining_already
15	**virus [✓]**	forecast [✓]	security [✓]	arid [✓]	budget_unfccc	developing_maintaining
16	**illnesses [✓]**	employment [✓]	solar [✓]	tree [✓]	arrangements_provide	clearer_development
17	**loss_lives [✓]**	fairness [✓]	power [✓]	livelihood [✓]	extra_finance	path_pursued
18	**epidemics [✓]**	prices [✓]	technology [✓]	agroforestry [✓]	decades_instability	rates_beyond
19	**deaths [✓]**	industries [✓]	carbon [✓]	resource_management [✓]	war_made	given_extremely
20	**loss_life [✓]**	economic_growth [✓]	energy [✓]	land_management [✓]	notable_made	economy_compromising
21	**mortality [✓]**	economic_development [✓]	ensembles	farming [✓]	work_parts	issue_underpins
22	**wellbeing [✓]**	low_carbon [✓]	world_today	productivity [✓]	giroa_mainstream	entire_social
23	**dara**	gdp [✓]	relies_directly	crops [✓]	estimate_nepa	concerns_continues
24	**ccsap**	cost [✓]	available_natural	livelihoods [✓]	napa_giroa	imf_estimate
25	**inhibitors**	needs [✓]	agreement_extensive	crop [✓]	economy_provision	giroa_health
26	**conditional_summary**	economic [✓]	description_fairness	resource [✓]	conviction_countering	provision_important
27	**droughts_likely**	resources [✓]	towards_lowering	biodiversity [✓]	commitment_countries	enable_rapid
28	**security_threat**	dataset	dangerous_levels	livestock [✓]	ncsa_submitted	developed_indc

Our approach to the keyATM was to fit these four topics, and to select six topics overall, which means that two additional topics were produced through the keyATM process. These can be seen in Table S2. While, we do not examine the two additional topics further in our analysis, it is worth noting that based on our interpretation of these key words and our reading of the NDCs, we identify the Other_1 topic as capturing a *developing countries* theme, and Other_2 as representing residual issues in the NDCs. We used the proportion of the *health* theme compared to the other five topics to produce our alternative measure of health engagement, the *Health Topic Proportion* (HTP). It is important to note that probabilistic topic models are based on inherently stochastic processes, and so there may be small changes in the topic distributions on repeated implementations. We ran several parallel chains and compared results. We find only minor differences, which were not substantively important.

Figure S6 shows countries' Health Topic Proportions in the form of a world map. As with the HES, we see a North-South divide in terms of levels of health engagement. Figure S7-S9 show countries' topic proportions for the other three fitted topics. We find evidence of this North-South divide for the agriculture and economy topic too, with developing countries discussing these topics more. For the energy topic, we also see a North-South

divided; however, here it is the higher income countries that discuss the topic more, and lower income countries that discuss it less.

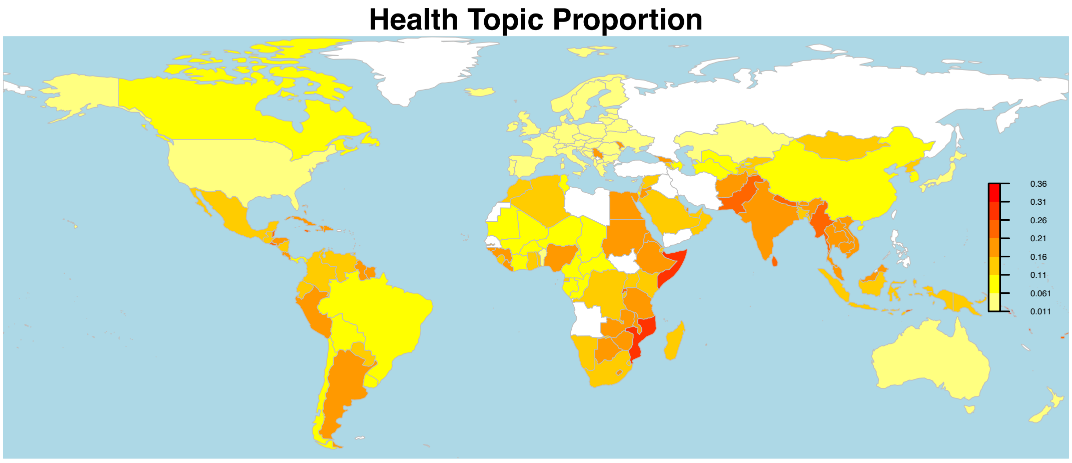

Figure S6. World map of health topic proportion produced through the topic model analysis.

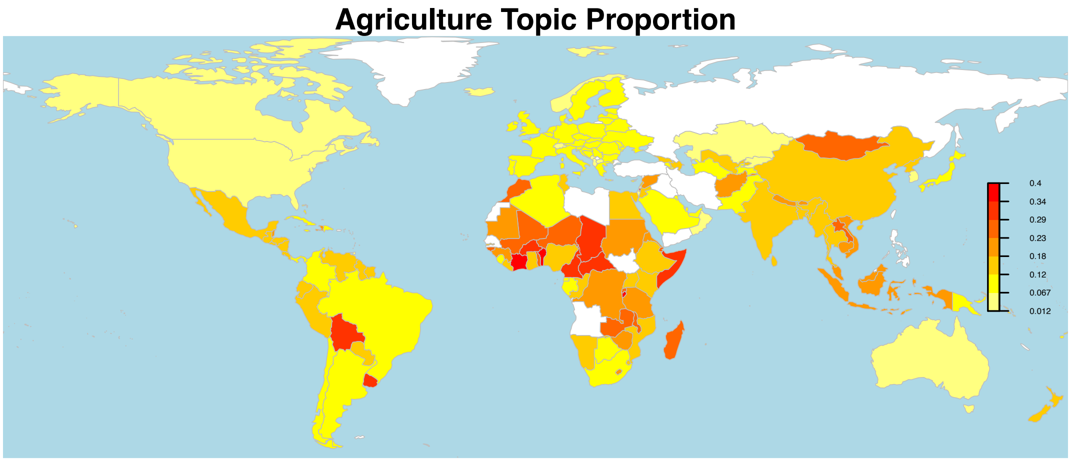

Figure S7. World map of health agriculture proportion produced through the topic model analysis.

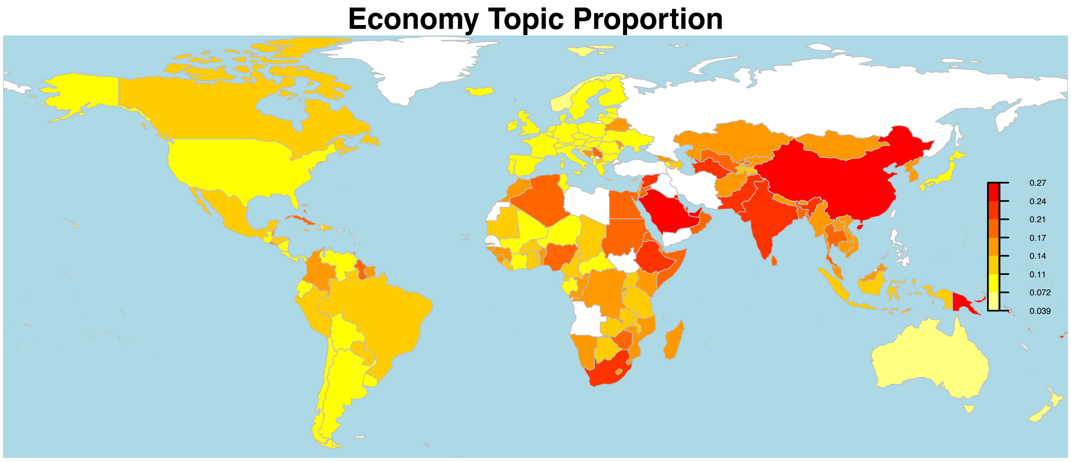

Figure S8. World map of economy topic proportion produced through the topic model analysis.

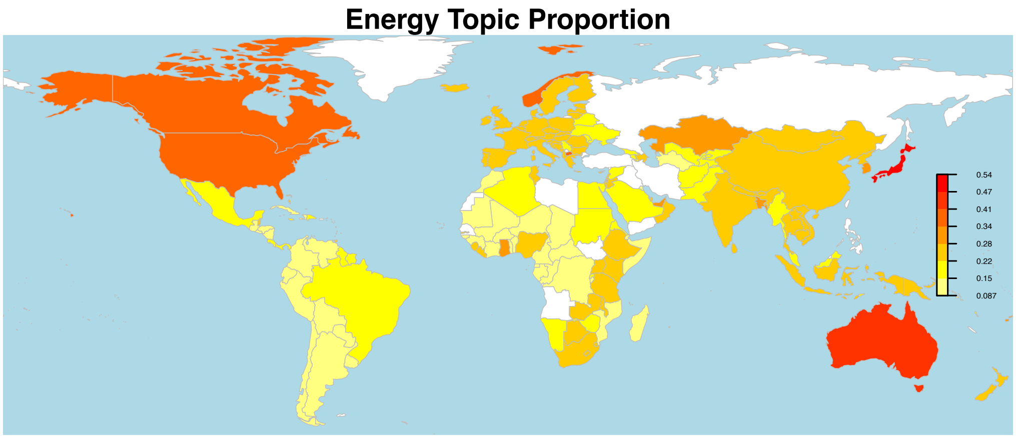

Figure S9. World map of energy topic proportion produced through the topic model analysis.

Figure S10 presents a density plot of the four topics where the area under the curve is normalised for each topic, enabling more direct comparisons. It further demonstrates the higher variation in how much countries discuss *health* compared to other topics. It also shows that fewer countries discuss *health* in detail compared to other topics, and that countries devote a higher proportion of their NDCs to discussing *energy* than other topics. Figure S11 shows an alternative representative of the distributions of the four fitted topics based on a histogram. It again shows the high number of NDCs with low topic proportions for health, as well as the significant variation in health topic proportions. It also shows that energy has the highest topic proportions in the NDCs.

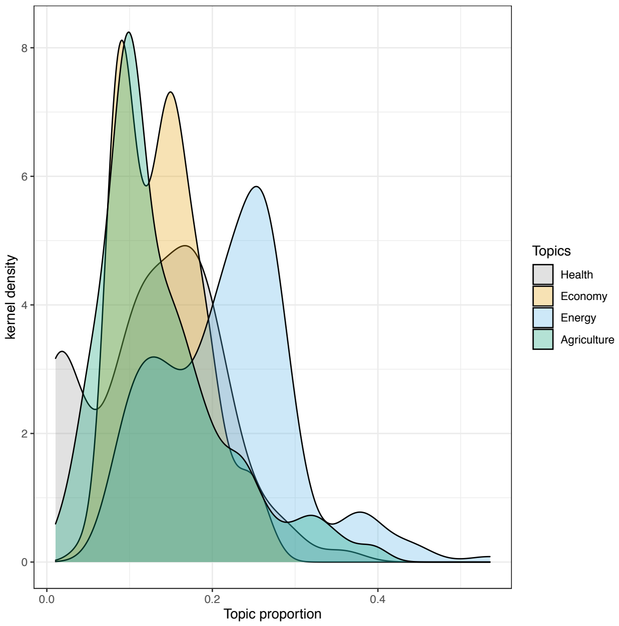

Figure S10. Density plot of topics in NDCs. Based on topic model analysis.

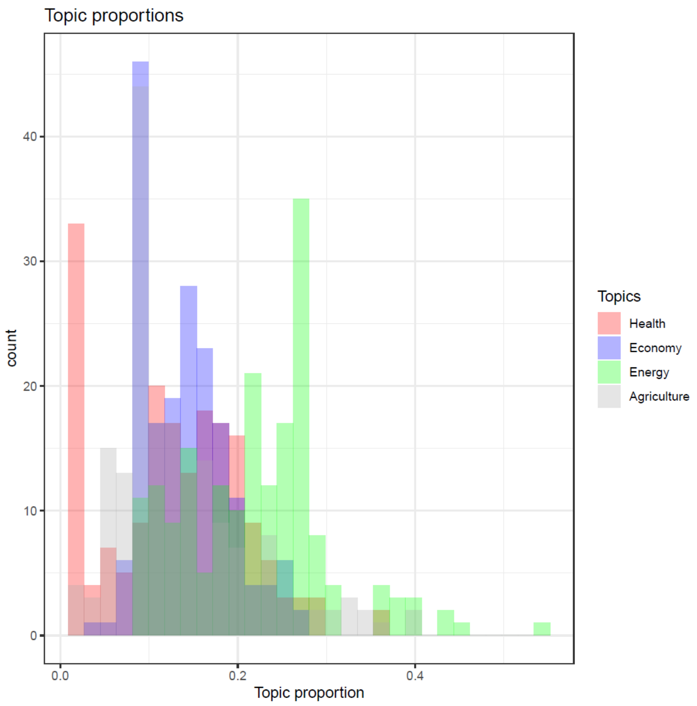

Figure S11. Histogram of topic proportions for four fitted topics.

We further compare usage of the four themes in the NDCs, by comparing the keyword proportions of each topic across the NDCs corpus. In other words, using our list of search terms for the four topics, we compare at how much these search terms feature in the NDCs. These are shown in Figures S12-S15. The figures show that health terms have the lowest proportions of the four topics, and energy has the highest. We also find that references to health focus on very few terms (e.g. *health* and *diseases*) compared to the other three topics, which have more key words with higher topic proportions.

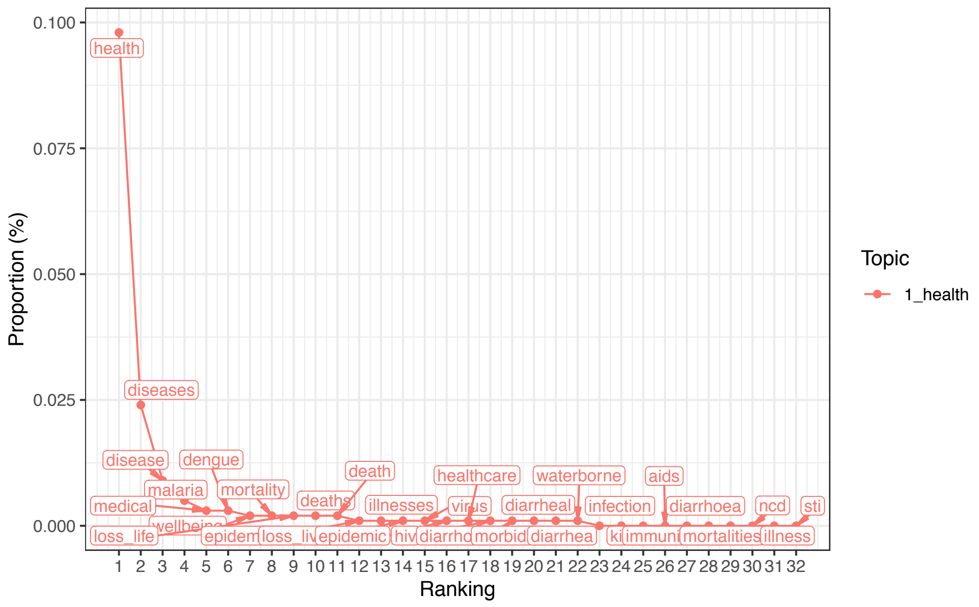

Figure S12. Proportions of search terms for *health* theme appearing in NDCs.

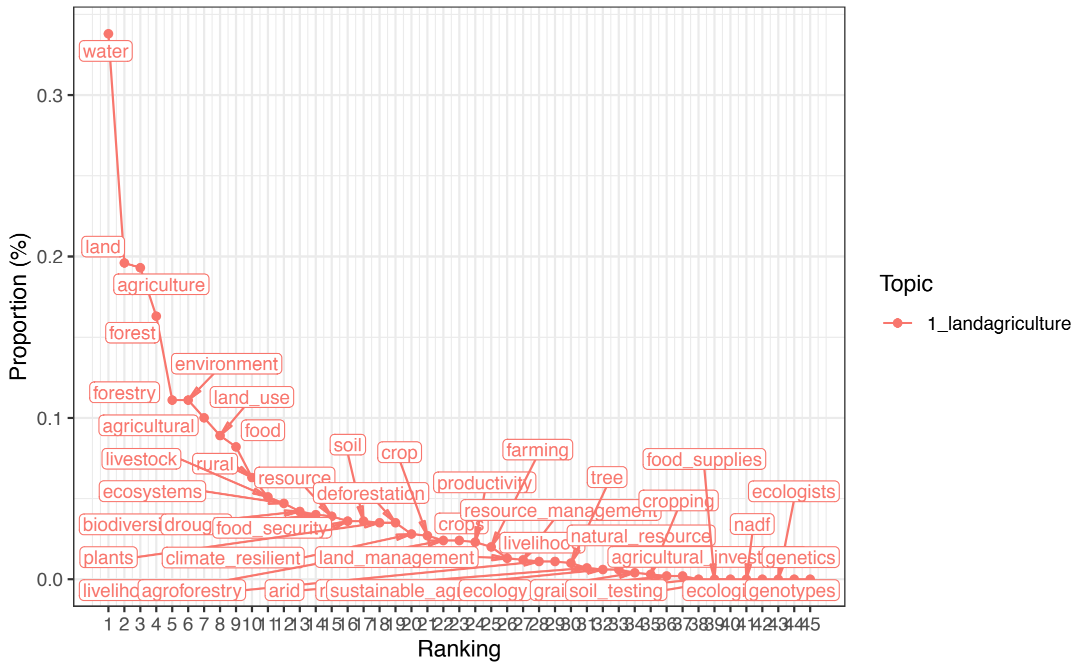

Figure S13. Proportions of search terms for *agriculture* theme appearing in NDCs.

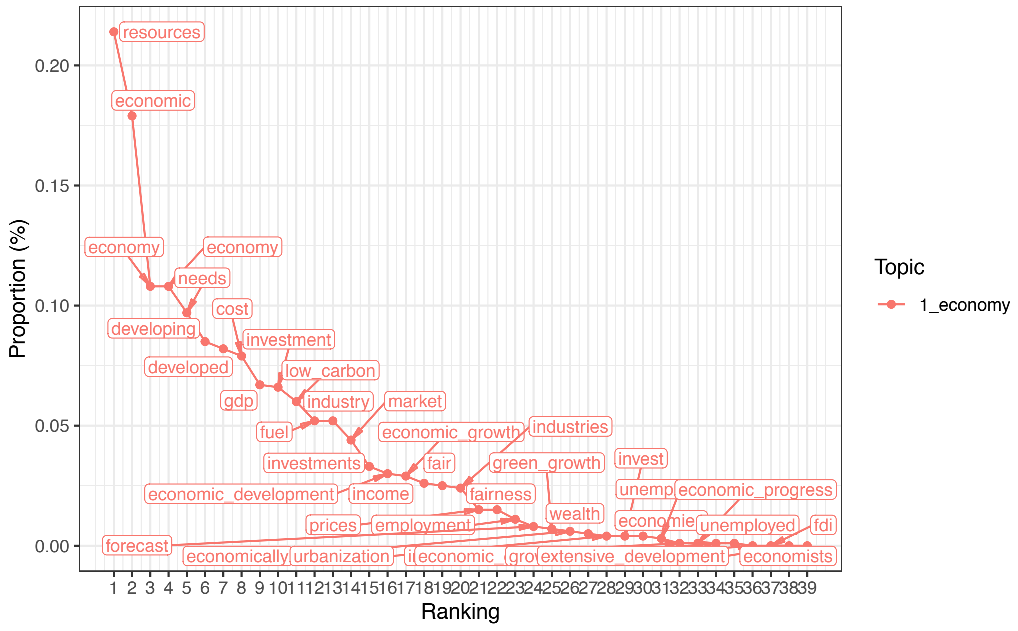

Figure S14. Proportions of search terms for *economy* theme appearing in NDCs.

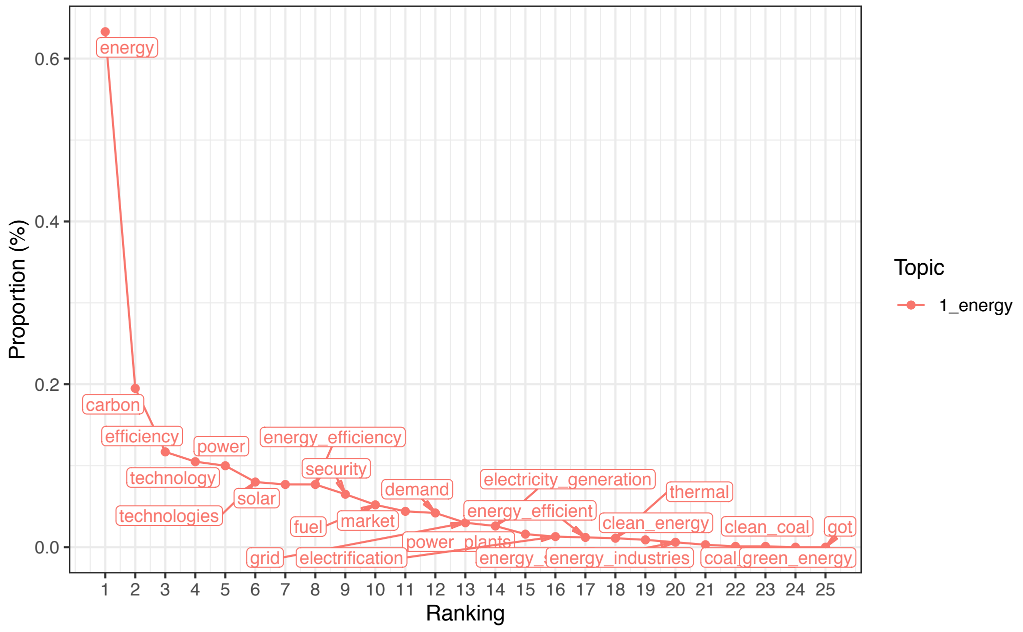

Figure S15. Proportions of search terms for *energy* theme appearing in NDCs.

In the main paper, we examine country characteristics associated with the HTP using a multivariate regression analysis. We do the same here for the other three fitted topics. The results are presented in Tables S3-S5.

Table S3: Association between country characteristics and the agriculture topic proportion. OLS regression with heteroscedasticity-robust standard errors.

	Coefficient (95% CI)	*p value*
Population (logged)	0.000 (-0.004 to 0.004)	0.896
SIDS	-0.029 (-0.051 to -0.006)	0.013
GDP per capita (logged)	-0.029 (-0.035 to -0.022)	0.000
Democracy	0.003 (-0.000 to 0.006)	0.095
Health expenditure (% of GDP)	-0.003 (-0.006 to -0.000)	0.026
Coal rents (% of GDP)	-0.010 (-0.042 to 0.021)	0.522
Temperature change	0.013 (-0.009 to 0.035)	0.238
Air pollution exposure	0.001 (0.000 to 0.023)	0.023
R^2	0.47	--
N	175	--

Table S4: Association between country characteristics and the economy topic proportion. OLS regression with heteroscedasticity-robust standard errors.

	Coefficient (95% CI)	*p value*
Population (logged)	0.002 (-0.002 to 0.005)	0.394
SIDS	0.069 (0.050 to 0.088)	0.000
GDP per capita (logged)	0.002 (-0.003 to 0.006)	0.430
Democracy	-0.006 (-0.009 to -0.004)	0.000
Health expenditure (% of GDP)	-0.001 (-0.003 to 0.001)	0.454
Coal rents (% of GDP)	0.016 (-0.001 to 0.033)	0.060
Temperature change	0.008 (-0.004 to 0.021)	0.199
Air pollution exposure	0.001 (0.000 to 0.001)	0.001
R^2	0.48	--
N	175	--

Table S5: Association between country characteristics and the energy topic proportion. OLS regression with heteroscedasticity-robust standard errors.

	Coefficient (95% CI)	*p value*
Population (logged)	0.002 (-0.005 to 0.009)	0.632
SIDS	0.019 (-0.015 to 0.052)	0.282
GDP per capita (logged)	0.024 (0.016 to 0.031)	0.000

Democracy	0.004 (-0.001 to 0.009)	0.104
Health expenditure (% of GDP)	0.003 (-0.002 to 0.008)	0.189
Coal rents (% of GDP)	0.012 (-0.005 to 0.029)	0.160
Temperature change	-0.013 (-0.039 to 0.012)	0.306
Air pollution exposure	0.000 (-0.000 to 0.001)	0.266
R^2	0.31	--
N	175	--

Tables S3-S5 show the results OLS regression analyses on each of the three additional fitted topics (agriculture, economy, and energy) with the country characteristics used in the regression analysis in the main paper. We find that GDP per capita and health expenditure (% GDP) have a positive and statistically significant association with the agricultural topic proportion (95% CI). For the economy topic, we find that SIDS (p=0.000), democracy (p=0.000), and air pollution exposure (p=0.001). It is worth noting that democracy is negatively associated with economy topic proportion. We find that GDP per capita is positively associated with the energy topic proportion (p=0.000). The variables that are significantly associated with each topic (including the health topic) at the 95% CI are shown in Figure S16 below.

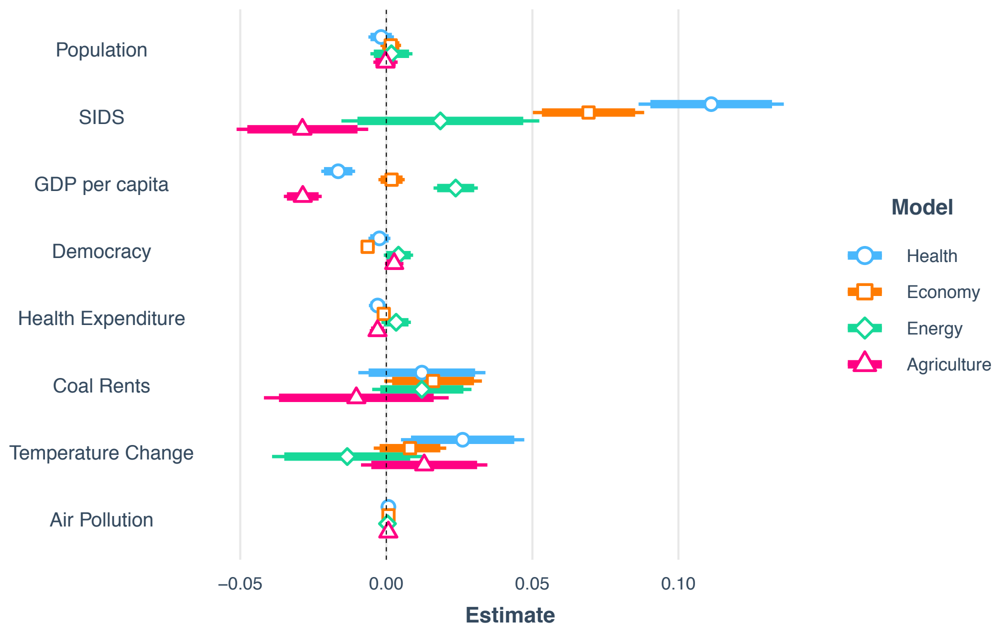

Figure S16. Marginal effects of country characteristics for each of the four topic proportions.

Control Variables Included in the Statistical Analysis

As we note in the main paper, we include the following variables in our multivariate regression models: size of country's population, GDP per capita (logged), SIDS, health spending (% of GDP), coal rents (% of GDP), population-weighted change in country's temperature, and exposure to ambient air pollution. Our choice of variables to include in the regression models was guided by three factors: (1) existing research; (2) data availability; and (3) high correlation between variables.

Existing research suggests that political, economic, and demographic factors generally influence the countries' governance. For example, it has long been argued that due to electoral competition, democracies are more responsive to their citizens than autocracies. There is some evidence to suggest this includes greater responsiveness in terms of health and health spending.[vii] There is a large amount of research that considers how levels of GDP per capita relate to both climate change and health, and as we explain in the paper previous research has found that the SIDS lead engagement on climate change and health in multilateral forum. The additional variables are included based on the expectation, derived from existing studies, that countries that spend more on health, profit less from fossil fuels, and are more exposed to the health impacts of climate change, are likely to engage more with such health impacts in their NDCs.

Our choice of variables, however, was also guided by data availability and the issue of high correlation – both of which are especially important as we conducted a cross-sectional analysis with a relatively small number of observations. Hence, our choice of variables was also based on maximizing our sample size. For a number of variables there is significant amounts of missing data for low income countries. Hence, we use coal rents (% of GDP) as a measure of the importance of fossil fuel revenues for the national economy because there is less country coverage for other fossil fuel revenues. Similarly, for more specific variables related to climate change and health, data was often only available for specific regions and countries, whereas the population-weighted temperature change and air pollution exposure measure had global coverage. Another issue that influenced our choice of variables was the high levels of correlation between variables, which leads to the problem of multicollinearity in the regression models. This was particularly related to the GDP per capita variable. We excluded several climate change (e.g. greenhouse gas emissions) and health variables (e.g. maternal mortality rate) because they were highly correlated with GDP per capita for our cross-sectional data.

We conduct additional analysis here of the main results related to the multivariate regression analysis using the HES as the outcome variable. As the length of NDC documents vary considerably, we consider whether our findings with the HES are influenced by the length of the documents. We do this by including an additional control variable in the regression model based on the total words in the NDC. We present the results in Table S7 below. The results show that are findings are not influenced by the inclusion of total words in the regression model with SIDS, GDP per capita, and coal rents remaining statistically significant (at the 95% CI).

Table S7: Association between country characteristics and health engagement in NDCs controlling for total words. OLS regression with heteroscedasticity-robust standard errors.

	Health Engagement Score	
	Coefficient (95% CI)	*p value*
**Total words**	0.000 (0.000 to 0.000)	0.000
**Population (logged)**	0.035 (-0.060 to 0.130)	0.473
**SIDS**	0.774 (0.249 to 1.299)	0.004
**GDP per capita (logged)**	-0.164 (-0.310 to 0.018)	0.028
**Democracy**	-0.136 (0.018 to 0.130)	0.130
**Health expenditure (% of GDP)**	0.019 (-0.069 to 0.106)	0.675
**Coal rents (% of GDP)**	-0.281 (-0.535 to -0.028)	0.030
**Temperature change**	0.383 (-0.056 to 0.821)	0.087
**Air pollution exposure**	0.003 (-0.010 to 0.017)	0.633
**R^2**	0.53	--
**N**	175	--

Additional Statistical Analysis with Alternative Outcome Measures

In addition to our HES and HTP measures of health engagement in the main paper, we use additional measures of health engagement to check the robustness of our findings. We use two additional health engagement measures: (1) the total count of health terms in the NDCs (based on the search terms provided in Table S1; and (2) the mentions of health terms as a percentage of all words in the documents. Figures S17 and S18 show the level of health engagement for countries based on these two measures.

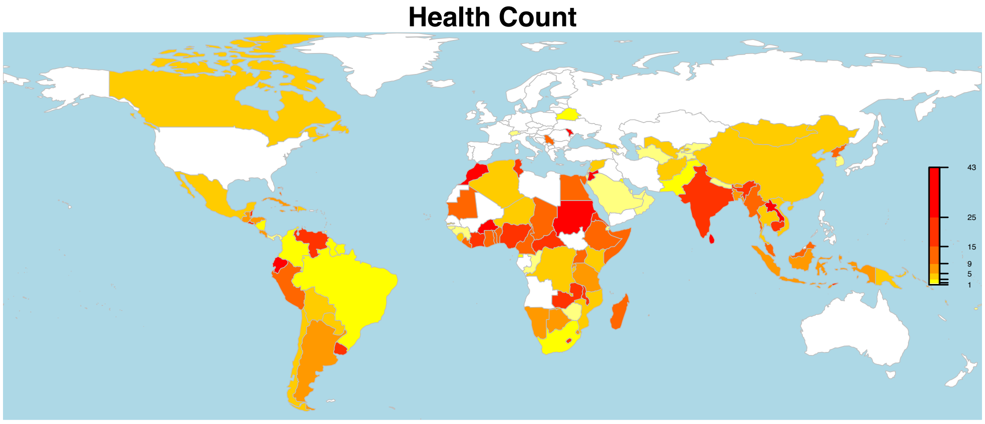

Figure S17. World map of total count of health terms in NDCs.

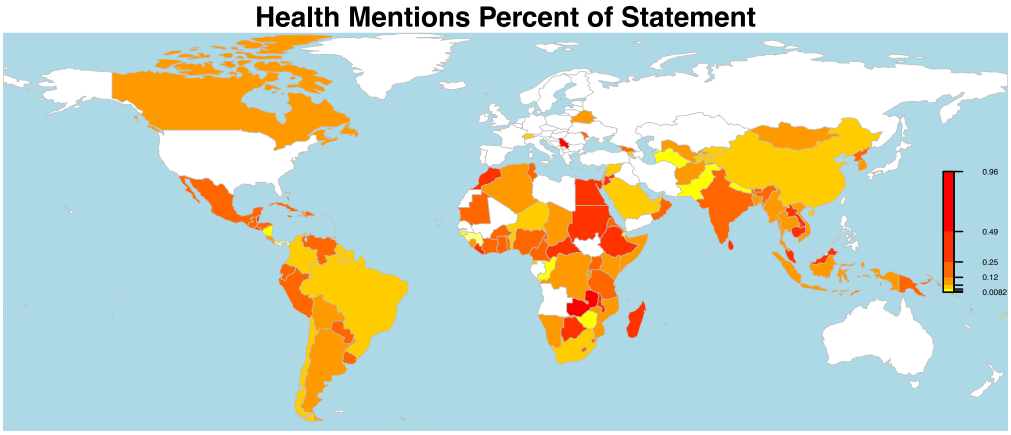

Figure S18. World map of percentage of health mentions in NDCs.

To examine the association of country characteristics with the health engagement measures, we conduct a multivariate regression analysis. As the total health count measure has a Poisson distribution (see Table S10), we use a Poisson regression. We use an OLS regression model for the percentage of health mentions measure. We present the results of the regression results with the two measures in Tables S8 and S9. The results in Table S6 show that when we use the total health count measure, we find that GDP per capita (p=0.000) and population-weighted temperature change (p=0.006) have a statistically significant association (at 95% level) with the total health count measure. With the percentage of health mentions measure (Table S9), we find that GDP per capita (p=0.000) has a negative association. This provides some evidence for the robustness of our findings regarding the relationship between GDP per capita and health engagement.

Table S8: Association between country characteristics and the total count of health terms. Poisson regression with heteroscedasticity-robust standard errors.

	Coefficient (95% CI)	*p value*
Population (logged)	0.041 (-0.068 to 0.149)	0.464
SIDS	0.412 (-0.121 to 0.944)	0.130
GDP per capita (logged)	-0.343 (-0.461 to -0.224)	0.000
Democracy	0.046 (-0.038 to 0.131)	0.283
Health expenditure (% of GDP)	-0.062 (-0.130 to 0.005)	0.071
Coal rents (% of GDP)	-0.452 (-0.968 to 0.063)	0.085
Temperature change	0.791 (0.232 to 1.350)	0.006
Air pollution exposure	0.003 (-0.009 to 0.015)	0.612
R^2	0.91	--
N	175	--

Table S9: Association between country characteristics and the percentage of health terms. OLS regression with heteroscedasticity-robust standard errors.

	Coefficient (95% CI)	*p value*
Population (logged)	0.003 (-0.004 to 0.010)	0.440
SIDS	0.040 (-0.013 to 0.092)	0.141
GDP per capita (logged)	-0.021 (-0.033 to -0.010)	0.000
Democracy	-0.002 (-0.009 to 0.005)	0.581
Health expenditure (% of GDP)	-0.005 (-0.011 to 0.001)	0.121
Coal rents (% of GDP)	-0.019 (-0.047 to 0.009)	0.181
Temperature change	0.036 (-0.020 to 0.091)	0.203
Air pollution exposure	-0.000 (-0.001 to 0.001)	0.743
R^2	0.123	--
N	175	--

We provide the complete summary statistics for all the variables including in the multivariate regression analyses (in main paper and appendix) in Table S10.

Table S10. Summary statistics for all variables including in multivariate regression analysis (N=175).

	**Mean (SD)**	**Minimum**	**Maximum**
**Any health mention**	0.726 (0.447)	0	1
**HES**	1.760 (1.565)	0	5
**HTP**	0.128 (0.079)	0.011	0.361
**Agriculture topic proportion**	0.135 (0.072)	0.012	0.389
**Economy topic proportion**	0.138 (0.049)	0.039	0.273
**Energy topic proportion**	0.218 (0.078)	0.087	0.535
**Total count of health terms**	6.240 (8.511)	0	43
**Percentage of health mentions**	0.108 (0.130)	0	0.958
**Population (logged)**	15.541 (2.185)	9.326	21.044
**SIDS**	0.206 (0.405)	0	1
**GDP per capita (logged)**	8.599 (1.476)	4.206	12.043
**Democracy**	6.973 (2.940)	0	10
**Health expenditures (% of GDP)**	6.828 (2.833)	1.749	17.862
**Coal rents (% of GDP)**	0.089 (0.402)	0	4.362
**Temperature change (population-weighted)**	0.771 (0.398)	-0.220	1.751
**Air pollution exposure**	24.279 (16.490)	5.700	94.300

Additional analysis comparing content of NDCs with higher and lower health engagement

In the main paper, we examine the differences between NDCs with higher and lower health engagement based on NLP *keyness* analysis. In this section, we provide some additional analysis, which provide further evidence of the wider differences in the content of NDCs that engage more with health compared to those that engage less with health. Figure S19 presents an additional bigram of statistically distinct words in NDCs for Health Engagement Scores 0 vs 1-5; Figure S20 shows the keywords distinguishing NDCs with HES 0-2 vs. HES 3-5; and Figure S21 shows the keywords distinguishing NDCs with HES 0-3 vs. HES 4-5. The results further demonstrate that NDCs with lower health engagement tend to have a narrower focus on emissions, energy use, and economic factors. In contrast, NDCs that engage more with health tend to have a broader focus on issues related to climate change vulnerability, mitigation, and adaptation. This is further demonstrated in Figures S22-S23, which presents word clouds that show the most frequent terms that appear in NDCs with higher engagement with health compared to lower health engagement. Figure S22 presents a word cloud comparing the most frequent words in NDCs with HES 0 vs 1-4, and Figure S23 shows HES 0-1 vs 2-5.

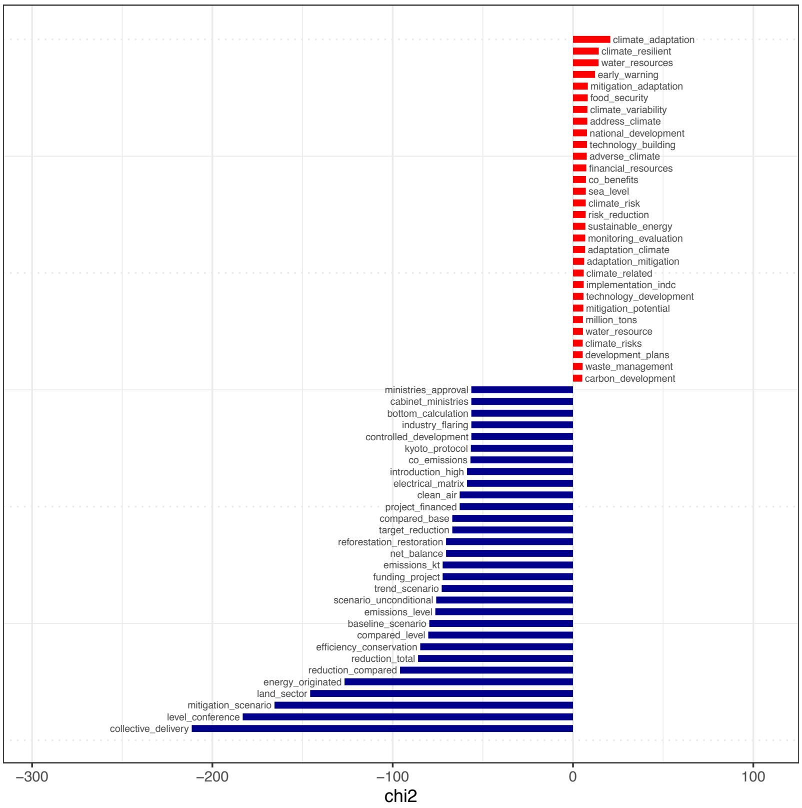

Figure S19. Keywords distinguishing countries that have no health engagement and those that engage with health. Bigram of statistically distinct words in NDCs with HES 0 (blue) vs. HES 1-5 (red).

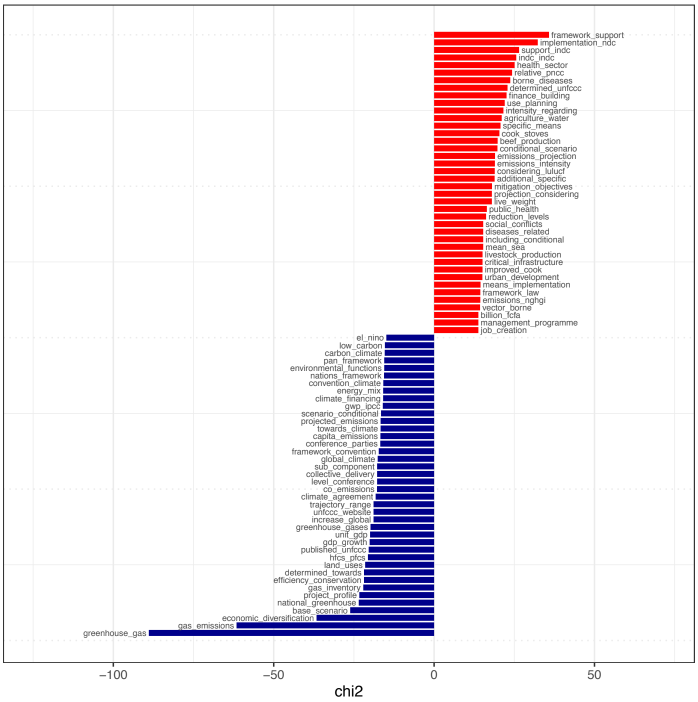

Figure S20. Keywords distinguishing countries that have lower health engagement and those that have higher health engagement. Bigram of statistically distinct words in NDCs for HES 0-2 (blue) vs. HES 3-5 (red).

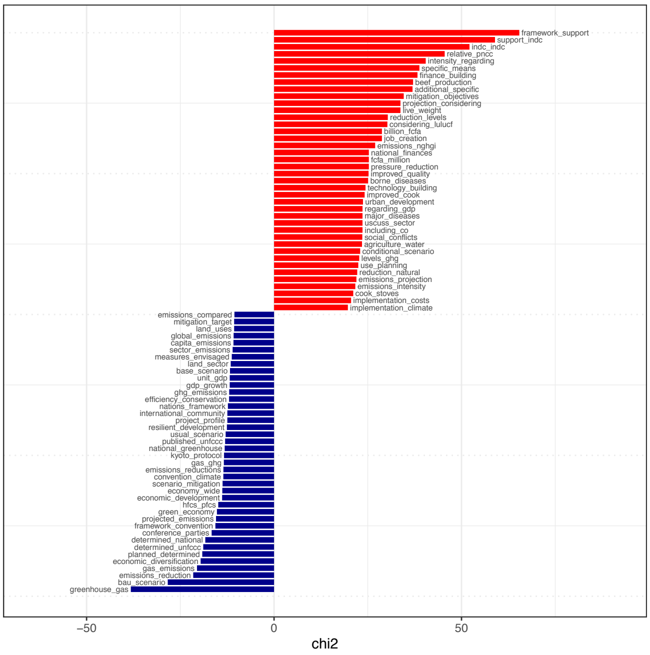

Figure S21. Keywords distinguishing countries that have lower health engagement and those that have higher health engagement. Bigram of statistically distinct words in NDCs for HES 0-3 (blue) vs. HES 4-5 (red).

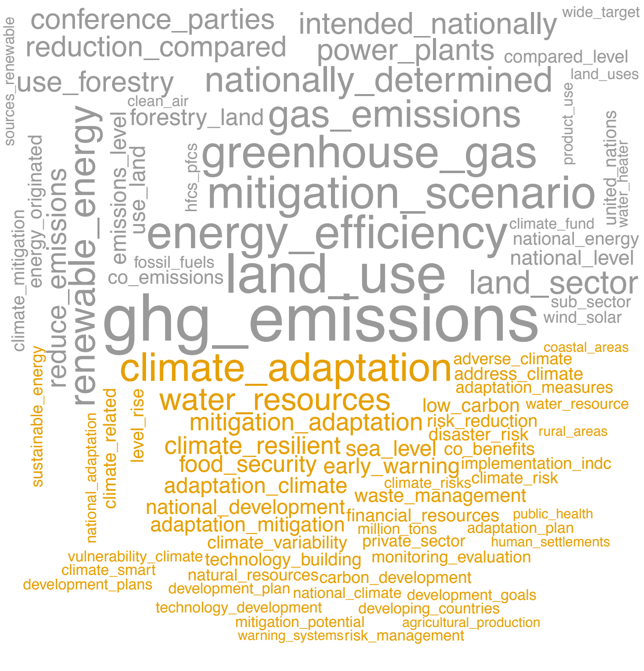

Figure S22. Word cloud showing most frequent terms in NDCs with HES 0 (top) vs HES 1-5 (bottom).

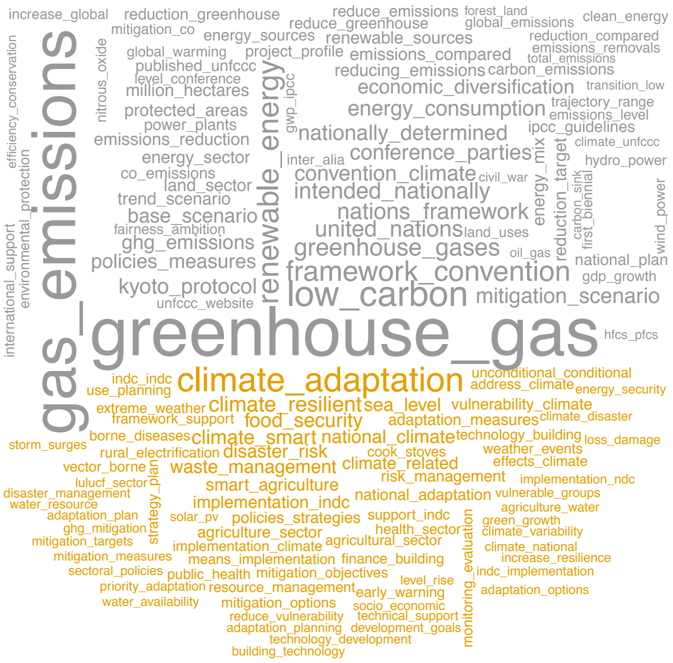

Figure S23. Word cloud showing most frequent terms in NDCs with HES 0-1 (top) vs HES 2-5 (bottom).

[i] UNFCCC [United Nations Framework Convention on Climate Change]. NDC Registry 2020. https://www4.unfccc.int/sites/NDCStaging/Pages/All.aspx (accessed May 15, 2020).

[ii] Watts N, Amann M, Arnell, N, et al. The 2019 report of The Lancet Countdown on health and climate change: ensuring that the health of a child born today is not defined by a changing climate. *The Lancet* 2019; **394**: 1836-1878.

[iii] Blei DM, Ng AY, and Jordan MI. Latent dirichlet allocation. *Journal of Machine Learning Research* 2003; **3**:993–1022.

[iv] Eshima S, Imai K, and Sasaki T. Keyword assisted topic models. *arXiv preprint arXiv:2004.05964* 2020.

[v] Jagarlamudi J, Daum'e H, and Udupu R. Incorporating lexical priors into topic models. *Proceedings of the 13th Conference of the European Chapter of the Association for Computational Linguistics:* 204-213.

[vi] Eshima S, Imai K, and Sasaki T. Keyword assisted topic models. *arXiv preprint arXiv:2004.05964* 2020.

[vii] Ross M. Is democracy good for the poor? *American Journal of Political Science* 2006; **50:** 860-874.
